# Better prognostic determination and feature characterization of cutaneous melanoma through integrative genomic analysis

**DOI:** 10.18632/aging.102099

**Published:** 2019-07-18

**Authors:** Xia Li, Yunpeng Cai

**Affiliations:** 1 Research Center for Biomedical Information Technology, Shenzhen Institutes of Advanced Technology, Chinese Academy of Sciences, Shenzhen 518055, P.R. China

**Keywords:** melanoma, heterogeneity, classification, better outcome, multi-omics

## Abstract

Melanoma is the most dangerous type of skin cancer and has highly heterogeneous features. Despite progress in melanoma classification, interpatient heterogeneity remains difficult to predict, especially in terms of long-term survival. Here, based on mRNA-seq, miRNA-seq and DNA methylation data from 447 cutaneous melanoma patients in the Cancer Genome Atlas, we performed integrative and single-dataset clustering analyses. A novel group of patients was identified, including 301 with better, 55 with poorer and 91 with intermediate prognoses. Immune genes were upregulated in the better prognostic group, and higher immune scores (representing a greater extent of immune cell infiltration into tumor tissues) were associated with better prognoses. Higher expression of 115 genes was determined to predict better outcomes. The better prognostic group also exhibited DNA hypomethylation, and immune pathways were enriched among the hypomethylated genes. Using exome-seq data from the same patients, we observed that the better prognostic group harbored the highest number of mutations. The mutational signature in the better prognostic group was associated with ultraviolet light exposure. These integrated investigations have potential therapeutic significance, as they clarify the molecular heterogeneity of cutaneous melanoma and enhance its classification.

## INTRODUCTION

Skin cancer is the most commonly occurring type of cancer. Nonmelanoma skin cancer, the most prevalent form, includes two main histological subtypes: basal cell and squamous cell carcinoma [1]. Melanoma, on the other hand, originates in melanocytes, the skin cells that produce melanin. The most common subtypes of melanoma are cutaneous and uveal [1]. Though melanoma represents only 4% of skin cancer cases, it exhibits the most aggressive, complex and heterogeneous features among skin cancers [2], and accounts for more than 75% of skin cancer-related deaths worldwide [3]. Over the past few decades, the incidence of cutaneous melanoma has risen by ~3% per year in the United States [4].

The molecular characteristics of both uveal [5] and cutaneous melanoma [6] exhibit internal heterogeneity, which is the main obstacle to personalized medicine, and is a major determinant of drug resistance [7]. There is thus an urgent need to classify cutaneous melanoma patients accurately and identify molecular markers to improve overall survival (OS). This will require extensive knowledge of the heterogeneity on the level of the genome, transcriptome and epigenome.

Genetic profiling has shown that the mutation rate of melanoma is the highest among all cancers [8]. Previous studies have divided melanomas into four groups based on the driver mutation: *BRAF*-mutant, *NRAS*-mutant, *NF1*-mutant and triple-wild-type [9]. However, subsequent studies showed that subtyping based on this genomic classification has little prognostic or diagnostic significance [9, 10]. On the other hand, a hierarchical clustering analysis based on gene expression identified three clusters of clinical relevance: “immune”, “keratin” and “microphthalmia-associated transcription factor (MITF)-low” [9]. In a study on 57 patients with Stage IV melanoma, unsupervised hierarchical clustering of gene expression data revealed four distinct subtypes, characterized by expression of immune-response, pigmentation-differentiation, proliferation or stromal-composition genes [11]. All four subtypes harbored *NRAS* and *BRAF* mutations, and the proliferative subtype was associated with poor survival. In another study, microarray-based gene expression profiling was performed on 41 multiple-melanoma biopsies from eight individual tumors [12]. These multi-region samples were pooled and classified into four subpopulations: high-immune (good prognosis), MITF-low proliferative (poor prognosis), MITF-high pigmentation (poor prognosis) and normal-like. Known mutations in *BRAF* and *NRAS* were either not detected or detected at very low levels in these samples. These studies indicated that gene expression profiles may be more informative than DNA mutations for classification.

DNA methylation arrays, which measure the methylation status of thousands of CpG sites across the genome, can also be used for cancer classification, possibly revealing additional complexity that cannot be captured at the expression level or through genetic profiling [13–15]. Methylation profiling has been widely used to delineate biologically relevant tumor subgroups [13, 16]. In a previous study, unsupervised k-means partitioning of whole-genome methylation profiles from 45 patients with Stage IIIC melanoma identified two classes of patients with significantly different OS [17].

Although dozens of classification schemes have been proposed to have clinical relevance, there is little agreement among these determinations, and the studies have used different patient groups, sample sizes and types of data. The heterogeneity among melanoma patients seems to depend on cues from the tumor microenvironment, but there is a lack of strong evidence on the mechanisms underlying interpatient heterogeneity, especially in terms of long-term survival. This prompted us to integrate gene expression and DNA methylation profiles in a larger study to identify prognostic subtypes with more favorable outcomes.

In the present study, we collected gene expression, miRNA expression and methylation data from 447 cutaneous melanoma patients in the Cancer Genome Atlas (TCGA), and employed these three genomic profiles in integrative and single-omics clustering analyses to identify patients with significantly different OS. Combining these data with gene expression data from three independent cohorts of melanoma patients, we identified and validated a list of genes that predicted better outcomes.

## RESULTS

### Identification of patients with long-term survival

We downloaded the DNA methylation, mRNA-seq and miRNA-seq profiles of 447 patients with cutaneous melanoma from the Genomic Data Commons (GDC). Of the patients, 171 (38.3%) were female and 276 (61.7%) were male (Supplementary Table 1). In total, there were 7 patients (1.6%) in Stage 0, 73 (16.3%) in Stage I, 128 (28.6%) in Stage II, 164 (36.7%) in Stage III, 23 (5.1%) in Stage IV and 52 (11.6%) of unknown clinical stage.

**Figure 1:**
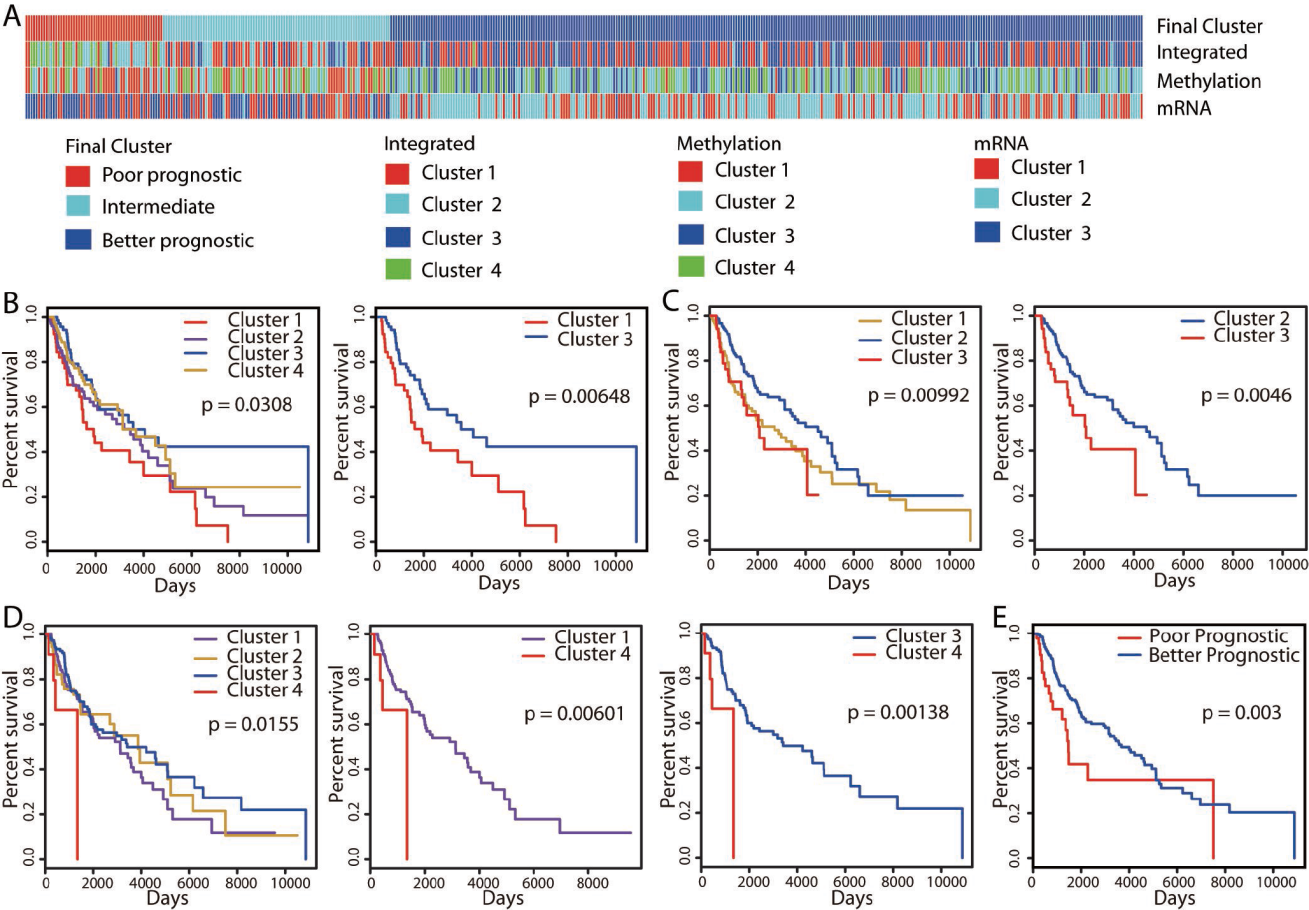
**Unsupervised clustering of 447 cutaneous melanoma patients and identification of the better prognostic group.** (**A**) Integrative clustering (“Integrated”) based on three omics datasets (methylation, mRNA-seq and miRNA-seq) identified four clusters. Clustering analyses of methylation and mRNA-seq data divided patients into four clusters (“Methylation”) and three clusters (“mRNA”), respectively. Kaplan-Meier OS curves are shown for the clusters identified by the “Methylation” (**B**), “mRNA” (**C**) and “Integrated” (**D**) classifications. Patients were pooled into the better prognostic group if they exhibited significantly (*P* < 0.01) better OS in at least two clusterings. A similar identification technique was used for the poorer prognostic group. The final clustering identified 301 patients with better prognoses, 55 patients with poorer prognoses and 91 patients with intermediate prognoses (**A**), and the associated Kaplan-Meier OS curve is shown in (**E**).

Using these three omics datasets, we performed an integrative unsupervised clustering analysis, which distinguished four clusters (Supplementary Figure 1) that were associated with differences in OS (Figure 1A, 1D). A total of 365 (365/447 = 81.7%) patients in both clusters 1 and 3 exhibited significantly better OS, and 20 (20/447 = 4.5%) patients assigned to cluster 4 displayed poor OS (hazard ratio [HR]: 4.01, 95% confidence interval [CI]: 1.38-11.71 [cluster 1 vs. cluster 4]; HR: 4.94, 95% CI: 1.67-14.57 [cluster 3 vs. cluster 4]; log-rank test, *P* < 0.01).

In addition to the clustering based on multiple omics datasets, we also performed subtype discovery using single datasets. We first performed unsupervised consensus clustering with the DNA methylation data. Ward linkage clustering and k-means clustering methods were applied, and both methods distinguished four clusters of patients (Supplementary Figure 2). However, only the clusters identified by the Ward linkage clustering analysis exhibited survival differences (Figure 1A, 1B; Supplementary Figure 2D). Therefore, we used this method for the consensus clustering analysis. Cluster 3 (n = 88, 19.7%) exhibited significantly better OS than cluster 1 (n = 73, 16.3%) (HR: 2.01, 95% CI: 1.20-3.35; log-rank test, *P* < 0.01).

When mRNA-seq data were used to evaluate gene expression, unsupervised consensus clustering analysis divided patients into three distinct clusters (Supplementary Figure 3). Similar results were obtained by k-means clustering (Supplementary Figure 3C), and the clusters from Ward linkage clustering were used for subsequent analyses. These clusters also displayed differences in OS, with significantly better survival in cluster 2 (n = 188, 42.1%) than in cluster 3 (n = 85, 19%) (HR: 2.06, 95% CI: 1.24-3.44; log-rank test, *P* < 0.01; Figure 1A, 1C). We also performed an unsupervised consensus clustering analysis on the miRNA-seq data, but found no obvious clustering structure.

The integrative clustering method identified more patients with better OS than the other two clustering methods. Among them, 82 (18.3%) and 169 (37.8%) patients were also marked by better OS in the methylation and mRNA-seq clusterings, respectively. An overlap of 52 patients (11.6%) was found between the two single-dataset clustering methods. In contrast, regarding patients with poorer OS, the methylation and mRNA-seq clusterings identified more patients than the integrative clustering. An overlap of 15 patients (3.4%) was observed between the two single-dataset clusterings. Only 1 patient in the methylation clustering and 17 patients (3.8%) in the mRNA-seq clustering were also marked by poorer OS in the integrative clustering. Thus, the patients exhibiting better/poorer OS in each clustering approach were different from those in the other two clusterings, so some patients would be missed if only one approach were used.

Therefore, we pooled all the patients with significantly different OS from the three clustering approaches to identify a final prognostic grouping of patients (Figure 1A). If both better and poorer OS were observed for a patient, the patient was pooled into the better prognostic group only if he/she exhibited significantly higher OS in at least two clusterings (Supplementary Figure 4). Similarly, a patient was pooled into the poorer prognostic group only if he/she exhibited significantly poorer outcomes in at least two clusterings. Ultimately, of the 447 cutaneous melanoma patients, we identified 301 patients with better prognoses, 55 patients with poorer prognoses and 91 patients with intermediate prognoses. In a survival analysis, the better prognostic group exhibited significantly better OS than the poorer prognostic group (HR: 2.07, 95% CI: 1.26-3.40; log-rank test, *P* < 0.01; Figure 1E).

### Immune-related genes were upregulated in the better prognostic group

**Figure 2:**
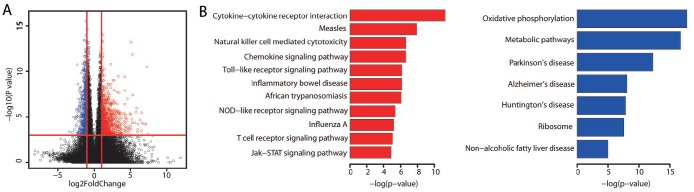
**Differentially expressed genes between the better and poorer prognostic groups.** (**A**) The significance of gene expression differences between the better and poorer prognostic groups. Each dot represents one gene. The *x* axis displays the gene expression difference as a log2-transformed fold-change. The *y* axis displays the significance as a -log10-transformed *P* value. The red vertical lines represent log2FoldChange values of -1 and 1, respectively. The horizontal red line represents a *P* value of 0.001. Genes were defined as differentially expressed if their absolute log2FoldChange values were greater than 1 and their *P* values were less than 0.001. A red dot indicates high expression, while a blue dot indicates low expression. (**B**) Bar plots display the significantly (adjusted *P* value < 0.01) enriched KEGG pathways for the upregulated (red) and downregulated (blue) genes identified by WebGestalt analysis. *P* values were adjusted by the method of Benjamini and Hochberg.

We then performed a differential gene expression analysis on the three prognostic groups. The expression of 1,635 genes differed significantly between the better and poorer prognostic groups (Figure 2A, *P* < 0.001). Among these genes, 980 were upregulated and 653 were downregulated in the better prognostic group. In addition, 90 genes were upregulated and 35 genes were downregulated in the better prognostic group compared with the intermediate prognostic group. No significant difference in gene expression was found between the poorer and intermediate prognostic groups.

To explore the potential biological significance of the differentially expressed genes, we examined whether any pathways in the Kyoto Encyclopedia of Genes and Genomes (KEGG) were enriched. The genes that were upregulated in the better prognostic group versus the poorer prognostic group exhibited significant enrichment in the immune system, while the downregulated genes were most significantly involved in metabolic pathways (Figure 2B, adjusted *P* value < 0.01; Supplementary Tables 2 and 3). There was no significant KEGG pathway enrichment for the genes that were differentially expressed between the better and intermediate prognostic groups.

### Higher immune scores were associated with significantly better OS

**Figure 3:**
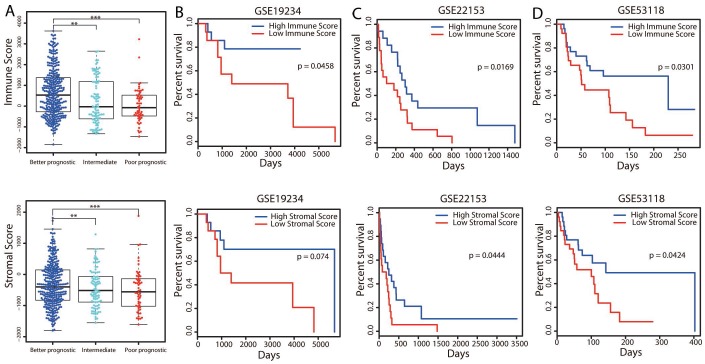
**Associations of immune and stromal scores with patients’ OS.** (**A**) The distribution of immune scores (top panel) and stromal scores (bottom panel) in the three groups. The boxplot displays that the immune/stromal scores were significantly higher in the better prognostic group and lower in the poorer prognostic group. **P < 0.01; ***P < 0.001. (**B**–**D**) Three independent cohorts of melanoma patients from GEO were separately divided into two groups (the top and bottom tertiles) based on their immune (top panel) and stromal (bottom panel) scores. Kaplan-Meier OS curves are shown for each dataset.

It was interesting that immune related pathways were upregulated in the better prognostic group compared with the poorer prognostic group, as several previous studies have reported the existence of an immune subtype [9, 11, 12]. We further analyzed the association of the immune system with better outcomes in cutaneous melanoma by determining patients’ immune scores and stromal scores. As shown in Figure 3A, the average immune score of the better prognostic group ranked the highest of the three groups, followed by those of the intermediate and poorer prognostic groups. Similarly, the rank order of the stromal scores was better > intermediate > poorer prognostic group. However, the immune and/or stromal scores were not associated with the survival groups in the integrative, methylation and mRNA-seq clustering approaches (Supplementary Figure 5). Thus, the immune and stromal scores correlated meaningfully with patients’ OS only in the pooled classification.

To validate the correlation of OS with the immune and stromal scores, we collected gene expression data from three independent cohorts comprising a total of 168 melanoma patients. The immune and stromal scores for these patients were predicted separately by an ESTIMATE algorithm, and the three cohorts of patients were divided into high- and low-score groups. Survival analyses revealed that patients in the high-score group, especially for immune scores, displayed better OS than those in the low-score group (Figure 3B–3D; log-rank test, *P* < 0.05). These results supported the association of the stromal score and especially the immune score with patients’ long-term survival.

### Identification and validation of prognostic genes

To explore the clinical relevance of the differentially expressed genes, we analyzed all 980 genes that were upregulated in the better prognostic group for their prognostic significance in terms of OS. High expression of 467 genes predicted significantly better OS in a log-rank test (Supplementary Table 4). To determine whether these genes were also of prognostic significance in other melanoma cases, we used the three independent cohorts for validation. Gene expression information on 295 of these genes was available for the three cohorts, and higher expression of 115 genes was validated to be linked to a significantly better prognosis (Supplementary Table 4).

**Figure 4:**
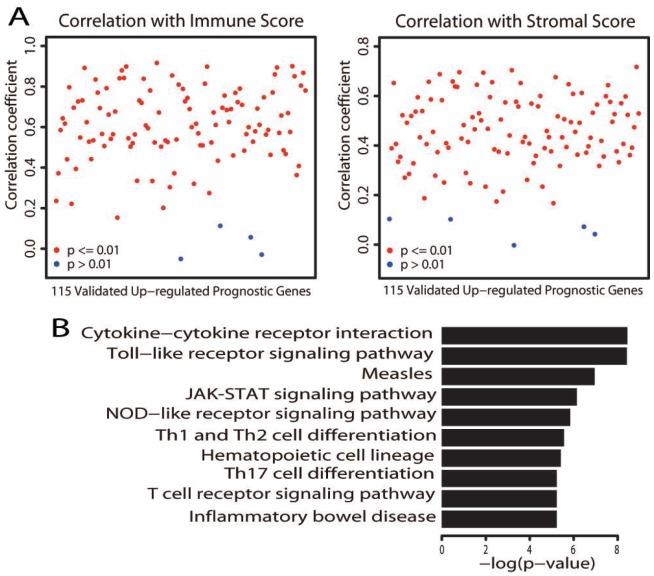
**Characterization of the validated upregulated prognostic genes.** (**A**) Correlation of the expression of the 115 validated upregulated prognostic genes with immune scores (left) and stromal scores (right). Each dot represents one gene. The *y* axis displays the correlation coefficient. Gene expression was deemed to correlate significantly with the immune/stromal score if the *P* value was less than 0.01 (red dot; a blue dot indicates *P* > 0.01). (**B**) Bar plots depict the significantly (adjusted *P* value < 0.01) enriched KEGG pathways.

We next assessed whether the expression of these validated prognostic genes was associated with the immune or stromal score. Significant positive correlations were found between patients’ immune/stromal scores and mRNA levels (Figure 4A, *P* < 0.01). We performed a KEGG pathway analysis to explore the functional significance of these genes. Similar to the aforementioned pathway enrichment analysis, these genes were significantly enriched in immune-related pathways (Figure 4B, adjusted *P* value < 0.01; Supplementary Table 5). These results provided further evidence that certain immune features are associated with a better prognosis.

### DNA hypomethylation was observed in the better prognostic group

**Figure 5:**
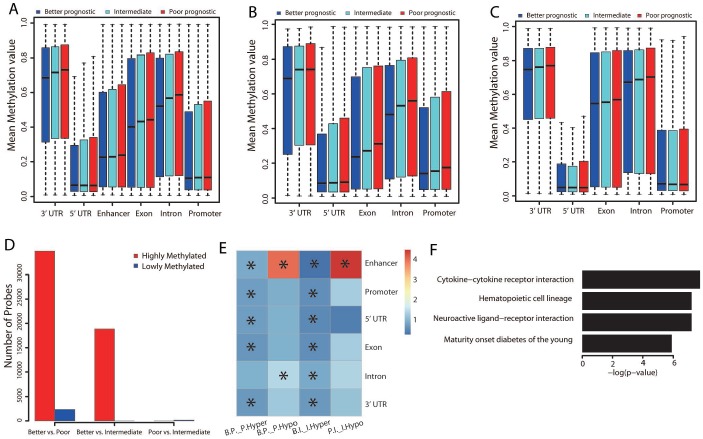
**Methylation differences among the three groups.** (**A**) Distribution of the mean beta values of probes across different genomic regions. The mean beta values of probes annotated to genes that were upregulated (**B**) or downregulated (**C**) in the better prognostic group versus the poorer prognostic group across different genomic regions are also shown. (**D**) Distribution of numbers of significantly methylated probes for each group comparison. (**E**) Genomic region enrichment of differentially methylated probes. We calculated the number of differentially methylated probes, along with the total number of probes on the bead array in each genomic region. Fisher’s exact test was used to test the enrichment. The heatmap displays the odds ratio, and the asterisks mark the significance (adjusted *P* value ≤ 0.001). “B.P._P.Hyper”: hypermethylated probes in the poorer versus the better prognostic group; “B.P._P.Hypo”: hypomethylated probes in the poorer versus the better prognostic group; “B.I._I.Hyper”: hypermethylated probes in the intermediate versus the better prognostic group; “P.I._I.Hypo”: hypomethylated probes in the intermediate versus the poorer prognostic group. (**F**) Significantly (adjusted *P* value < 0.01) enriched pathways of the hypomethylated genes in the better versus the poorer prognostic group.

To determine whether there were DNA methylation differences across the three prognostic groups, we performed further analyses by genomic region locality. The better prognostic group displayed the lowest methylation level, while the poorer prognostic group had the highest methylation level across all genomic regions (Figure 5A; Student’s *t* test, *P* < 0.001).

Three methyltransferases, DNMT1, DNMT3A and DNMT3B, establish and maintain the DNA methylation level. Thus, the expression of the genes encoding these enzymes was compared among the three groups. *DNMT1* expression did not differ significantly, but both *DNMT3A* and *DNMT3B* levels were marginally lower in the better prognostic group than in the other groups (*DNMT3A*: *P* = 0.02 [better vs. poorer], *P* = 0.04 [better vs. intermediate]; *DNMT3B*: *P* = 0.04 [better vs. poorer], *P* = 0.08 [better vs. intermediate]; Student’s *t* test).

We also extracted probes annotated to the differentially expressed genes between the better and poorer prognostic groups, and categorized them according to their locality on different genomic regions. We then calculated the mean beta value for each category of probes across the three groups. All the intragenic and promoter regions of the upregulated genes displayed the lowest methylation level in the better prognostic group (Figure 5B). In particular, the methylation level of the promoter regions was significantly lower in the better prognostic group than in the other two groups (Figure 5B; Student’s *t* test, *P* < 0.001). This indicated that the increases in gene expression resulted from reduced methylation of the gene promoters. In contrast, no obvious methylation differences were found in the downregulated genes across the three groups (Figure 5C). Thus, some other regulatory mechanism seemed to be responsible for their downregulation.

Differential methylation analysis revealed significant differences in the methylation of 37,164 probes between the better and poorer prognostic groups (Figure 5D). The majority of probes (34,816/37,164 = 93.7%) displayed significant hypomethylation in the better prognostic group, while 2,351/37,164 (6.3%) displayed hypermethylation in this group. Compared with the intermediate group, 18,868 probes were significantly hypomethylated in the better prognostic group, and 160 probes were hypermethylated in the poorer prognostic group. These results were consistent with the aforementioned observation that the rank order of methylation levels was poorer > intermediate > better prognostic group.

Interestingly, further analysis of these significantly methylated probes by genomic region revealed that almost all genomic regions were hypomethylated in the better prognostic group compared with the other two groups (Figure 5E). This was consistent with the negative correlation between the expression and methylation of upregulated genes across genomic regions. However, the methylation level of enhancer regions was remarkably greater in the better prognostic group than in the poorer prognostic group (Figure 5E). Hypermethylation was also observed in the enhancer regions of the poorer versus the intermediate prognostic group. This indicated that the hypermethylation of enhancer regions may have inhibited gene expression in the better prognostic group.

The functions of the differentially methylated genes were also examined by KEGG pathway analysis. Only the genes that were hypomethylated in the better versus the poorer prognostic group (n = 5,162) displayed significant pathway enrichment. Interestingly, immune-related pathways were significantly hypomethylated (Figure 5F, adjusted *P* value < 0.01; Supplementary Table 6).

### The mutation burden was higher in the better prognostic group

**Figure 6:**
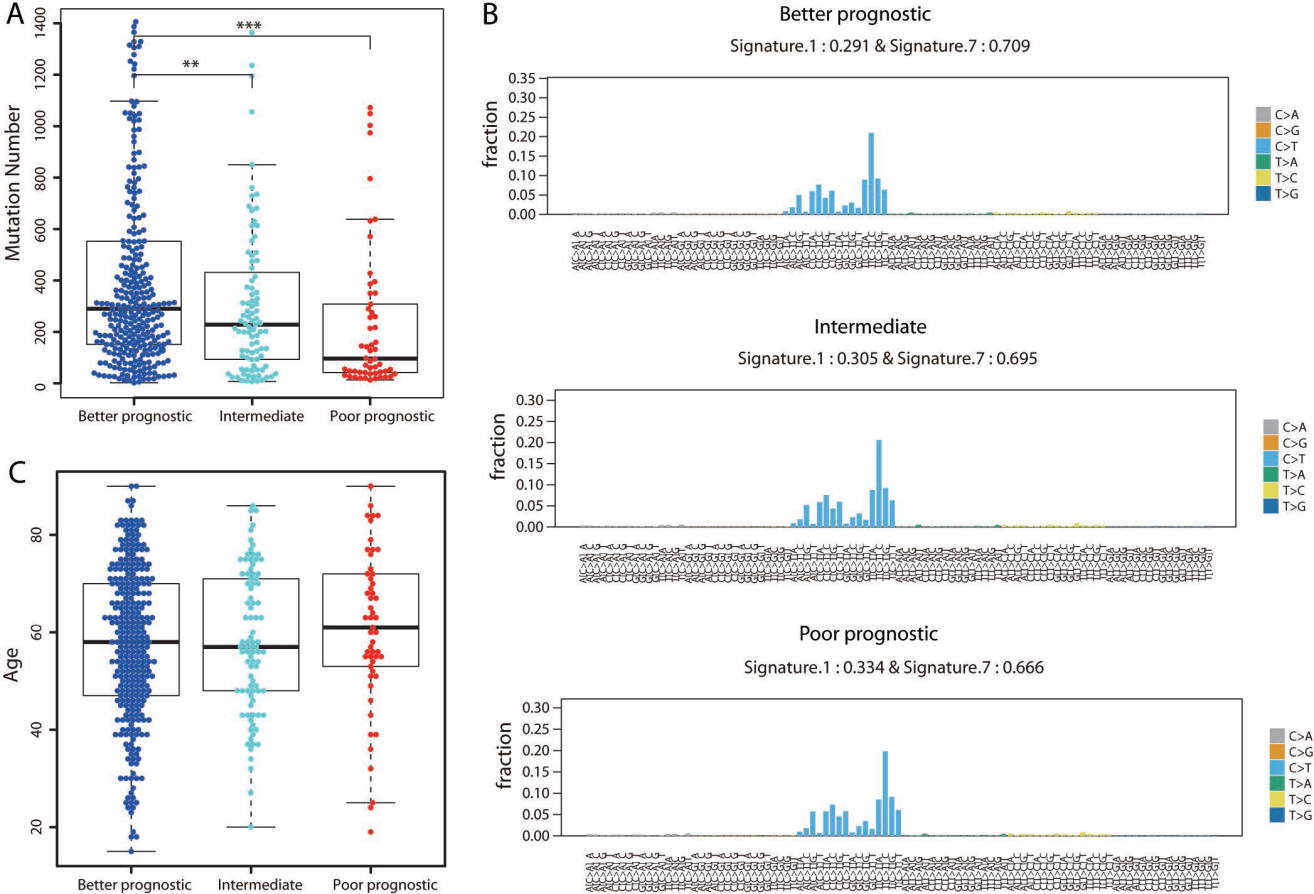
**Assessment of mutational differences among the three groups.** (**A**) The total number of nonsynonymous mutations is shown for each group. The *P* value was calculated by a two-sided Student’s *t* test. **P < 0.01; ***P < 0.001. (**B**) The 96-trinucleotide mutational spectra of mutations in the better (top panel), intermediate (middle panel) and poorer (bottom panel) prognostic groups were inferred by deconstructSigs. The fraction of mutations found in each trinucleotide context is displayed. Mutational Signatures 1 (associated with age) and 7 (associated with ultraviolet light exposure), together with the weights contributing to each group, are shown above each figure. (**C**) Distribution of ages in the three groups.

We then investigated whether there were differences in the mutation load across the three prognostic groups by calculating the number of nonsynonymous mutations. Surprisingly, the better prognostic group harbored the highest number of mutations, while the poorer prognostic group harbored the lowest number of mutations (Figure 6A; Student’s *t* test, *P* < 0.001). Of the known melanoma driver mutations (*BRAF*, *NRAS* and *NF1*), only *BRAF* was mutated significantly more frequently in the better than in the poorer prognostic group (162/301 vs. 16/55; Fisher’s exact test, *P* = 0.001).

We also extracted the mutational signature for each of the three groups separately to determine the contribution of a given mutational signature to an individual group. The overall mutational spectra were similar, with very strong enrichment of Signature 7, followed by Signature 1 substitutions (Figure 6B). Signature 7 has been found predominantly in skin cancers, and is likely due to ultraviolet light exposure. The weight assigned to Signature 7 was highest in the better prognostic group and lowest in the poorer prognostic group, in agreement with the notion that ultraviolet light causes mutations in DNA. In contrast, the age-associated signature (Signature 1) exhibited the highest weight in the poorer prognostic group and the lowest weight in the better prognostic group. Indeed, the poorer prognostic group included the oldest patient population (mean age: 60.55 years, Figure 6C); the average ages of the better and intermediate prognostic groups were 57.67 and 57.96 years, respectively. These results demonstrated that the temporal dynamics of mutagenesis contributed to the differences among these groups.

## DISCUSSION

Melanoma is highly heterogeneous on the genetic, expression, and epigenetic levels [18]. Rapid advancements in understanding this heterogeneity have enabled the molecular classification of melanoma and personalized medicine for its management [9–12, 17]. Despite this considerable progress, the underlying mechanisms driving interpatient heterogeneity, especially in terms of long-term survival, have remained unclear.

In the present study, we performed integrative and single-dataset clustering analyses of gene expression, miRNA expression and methylation data from 447 cutaneous melanoma patients. We identified a novel group of 301 patients with significantly better prognoses and 55 patients with poorer prognoses. The better prognostic group was characterized by high immune gene expression, high immune scores, DNA hypomethylation, a high mutation burden and a mutation signature associated with ultraviolet light exposure, while the poorer prognostic group exhibited the opposite characteristics.

Previous profiling studies have mainly included melanoma patients at later clinical stages [11, 17], whereas the present study included patients across all clinical stages, enabling more patients with similar molecular profiles to be clustered. In addition, unlike previous classifications based on either gene expression or DNA methylation data [9–12, 17], our classification was based on both integrative clustering and single-dataset clusterings, enabling us to decipher a much broader spectrum of interpatient heterogeneity. Moreover, to our knowledge, this was the largest sample size used for classification, and the results were applicable for a wide range of patients.

Our data corroborated the results of several other studies demonstrating the existence of an immune subtype. Melanoma cells are highly plastic and able to adapt quickly to changing microenvironmental conditions, such as the oxygen level and the immune cell composition [7]. These cues from the surrounding microenvironment activate intracellular molecular changes, resulting in tumor heterogeneity. The immune gene enrichment in the better prognostic group may have been influenced by the microenvironment.

In addition to detecting greater expression of immune genes in the better prognostic group, we found for the first time that higher immune scores were associated with better prognoses in cutaneous melanoma. These results were validated in an extension study on three independent cohorts of melanoma patients. Patients with higher immune scores exhibited better OS, suggesting that the immune score could be applied for the classification of cancer patients. Indeed, a previous report also raised this possibility [19]. Furthermore, in a very recent study, the immune score was determined to be a reliable estimate of the risk of colon cancer recurrence, and was thus implemented as a new component of cancer classification [20]. Further efforts to divide patients accurately based solely on their immune scores should enhance the area of cancer classification.

We also found that the better prognostic group displayed the lowest level of DNA methylation. Moreover, the expression of upregulated genes in the better prognostic group correlated negatively with their methylation levels, possibly due to the hypomethylation of their promoters. Immune pathways were also hypomethylated. Therefore, DNA hypomethylation greatly distinguished patients with better prognoses.

Two DNA methyltransferase genes, *DNMT3A* and *DNMT3B*, were downregulated in the better prognostic group, though not significantly so. Interestingly, previous studies have indicated that ultraviolet B radiation can enhance DNA hypomethylation by inhibiting the catalytic activity of DNMT1 [21, 22]. However, both DNA hypermethylation and hypomethylation have been reported in skin tumors exposed to ultraviolet radiation [23, 24], indicating possible methylation heterogeneity. In our data, the better prognostic group exhibited hypomethylation, while the poorer prognostic group exhibited hypermethylation. In addition, the mutational signature in the better prognostic group was associated with ultraviolet light exposure, while this signature was assigned the lowest weight in the poorer prognostic group. The reduced expression of *DNMT3A* and *DNMT3B* in the better prognostic group may have been responsible for the hypomethylation of genes in response to ultraviolet exposure. However, previous studies have also indicated that DNA methylation is strongly associated with histone acetylation upon ultraviolet skin exposure [23, 25]. This correlation is poorly understood and requires deeper investigation.

A few previous studies have evaluated ultraviolet light exposure and survival among melanoma patients. In a population study of cutaneous melanoma patients based on self-reported personal behavior in the sun, an independent inverse association was detected between the presence (versus absence) of solar elastosis (a histologic indicator of cutaneous sun damage) and disease-specific mortality (HR 0.40, 95% CI: 0.20-0.80) [26]. Another study also demonstrated that solar elastosis was associated with a better prognosis of melanoma [27], supporting ultraviolet light exposure as a protective prognostic factor. In a large multicenter cohort study with detailed sun exposure data, a sunburn within ten years of a melanoma diagnosis was found to reduce the HR of death (HR 0.27, 95% CI: 0.09-0.85) [28]. These results are in line with the results of the present study, suggesting that ultraviolet light exposure is associated with increased survival in patients with cutaneous melanoma.

The mechanisms whereby ultraviolet light exposure enhances the survival of melanoma patients remain speculative. The proposed mechanisms include increased vitamin D, nitric oxide [29] and melanin production, altered DNA damage-repair mechanisms [26, 30] and *BRAF* mutations [31]. Sun exposure is the primary source of vitamin D, and high vitamin D levels have been associated with reduced risks of cancer and overall mortality [32, 33]. Interestingly, there is evidence that certain genes in the vitamin D signaling pathway promote DNA demethylation [34], which may also account for the hypomethylation we observed in the better prognostic group.

A surprising finding of this study was that the mutational burden was the highest in the better prognostic group. We propose that this may have been due to ultraviolet light exposure. Consistently, in a previous study examining the relationship between the mutational burden and the outcomes of immunotherapy-treated patients with diverse cancers, a higher mutational burden was associated with better outcomes [35]. A higher number of mutations could generate a greater number of neo-antigens to be recognized by the immune system. The age of the patients did not seem to be associated with their mutational burden, because the patients in the poorer prognostic group were the oldest. Notably, older melanoma patients have been reported to have poorer prognostic outcomes because they have a higher incidence of developing other diseases [36]. Furthermore, being greater than 60 years old may be associated with having more aggressive histological features [37]. These reports support the rationality and biological meaningfulness of our classification and feature characterization.

In summary, we provided a novel and reasonable classification scheme for cutaneous melanoma. By integrating multiplatform data, we systematically characterized the molecular features of different subgroups, especially the better prognostic group. This study has enhanced our understanding of the mechanisms contributing to heterogeneity among cutaneous melanoma patients. Furthermore, the prognostic genes we identified and validated have potential applications in biomarker development and personalized medicine.

## METHODS

### Data source

We downloaded gene expression data (reads per kilobase per million mapped reads [RPKM] values) based on mRNA-seq; miRNA expression data based on miRNA-seq; methylation data (Illumina Human Methylation 450 platform, beta values) and clinical information for 447 skin cutaneous melanoma patients from TCGA under the GDC (https://portal.gdc.cancer.gov/) (April 12, 2018). Of the 470 samples available in the GDC, 447 had data for both transcriptome and DNA methylation profiling, and were used in subsequent analyses.

For validation, microarray expression profiles were obtained from the National Center for Biotechnology Information Gene Expression Omnibus (NCBI GEO, https://www.ncbi.nlm.nih.gov/geo/). The accession numbers for the three cohorts were GSE19234, GSE22153 and GSE53118.

### Clustering of molecular data for all cutaneous melanoma cases

In total, 485,577 probes were used to explore the DNA methylation profiles on the genomic scale. Beta values between 0 and 1 were used to represent the relative methylation level, which was measured as the ratio of the methylated probe intensity to all the methylation probe intensities. The top 10% most variable probes (those displaying the highest median absolute deviation across beta values) were included in consensus clustering analysis, which was performed with the widely used R package ConsensusClusterPlus [38].

The following settings were used in the consensus clustering: number of resamplings = 1000; pItem = 0.90 (resampling frequency samples); pFeature = 0.90 (resampling frequency); Pearson distance metric; Ward linkage clustering method. Consensus matrices were analyzed for the number of clusters *k* from 2 to 6, and a cumulative distribution function (CDF) was constructed for each *k*. The optimal number of clusters was determined from the CDFs and consensus matrices. The purpose of the consensus matrix plots was to find the “cleanest” cluster partition, while the purpose of the CDFs was to find the *k* at which the distribution reached an approximate maximum. The most robust result was found with a four-cluster solution. Alternatively, the k-means clustering method was used, and four clusters were identified.

For the mRNA-seq data, the RPKM values were log2-transformed. Similarly, the miRNA expression values (reads per million miRNAs mapped) were log2-transformed. The top 20% most variable genes and miRNAs (those with the highest median absolute deviation across all 447 patients) were separately retained for clustering. The number of clusters *k* from 2 to 6 was analyzed by ConsensusClusterPlus with the same parameters used for methylation clustering. The most robust result for the mRNA-seq data was found with a three-cluster solution, whereas no obvious clustering structure was found for the miRNA-seq data.

An integrative clustering analysis was conducted on all three datasets by a new method, moCluster [39], which performed robustly with a fast computation time. Gap statistics with respect to 1 to 12 clusters were analyzed, and a four-cluster structure was found to be the optimal choice.

### Analysis of differentially expressed genes and gene function

The mRNA-seq raw reads counts for 60,488 genes were downloaded from TCGA under the GDC (April 12, 2018). The DESeq [40] package in R software was used to identify differentially expressed genes. *P* values were adjusted by the method of Benjamini and Hochberg. A gene was defined as differentially expressed if the absolute value of the log2FoldChange was greater than 1 and the adjusted *P* value was less than 0.001.

Gene function analyses of all the differentially expressed genes were performed with WebGestalt [41] and the Database for Annotation, Visualization and Integrated Discovery (DAVID) [42]. In WebGestalt, overrepresentation enrichment analysis was selected as the method of interest, and genome protein-coding was selected as the reference set. *P* values were adjusted by the method of Benjamini and Hochberg. A pathway was considered to be significantly enriched if the adjusted *P* value was less than 0.01.

### Immune score and stromal score analyses

Based on gene expression data, immune scores and stromal scores were predicted by the ESTIMATE algorithm [43]. In general, the immune score represents the level of immune cell infiltration, while the stromal score represents the presence of stromal cells in tumor tissue. Two-sided Student’s *t* tests were used to evaluate the differences in immune or stromal scores between groups. Pearson’s correlation analysis was performed to assess the association of the immune or stromal score with gene expression across patients. A two-sided *P* value less than 0.01 was considered statistically significant.

### Differential methylation analysis

For each methylation probe, log2 transformation was performed to evaluate the methylation intensity ratio. The significance of differences in probe methylation between subgroups was determined with the Samr [44] package in R software. Probes with fold-changes greater than 1.25 and *q* values less than 0.01 were deemed to be highly methylated (hypermethylated), while those with fold-changes less than 0.8 and *q* values less than 0.01 were deemed to be hypomethylated.

Genes were defined as differentially methylated if their promoters contained differentially methylated probes. We excluded genes with promoters harboring both hypermethylated and hypomethylated probes. Differentially methylated genes were functionally analyzed with WebGestalt and DAVID. *P* values were adjusted by the method of Benjamini and Hochberg. A pathway was considered to be significantly enriched if the adjusted *P* value was less than 0.01.

### Genomic region distribution

Genomic regions including the 5’ untranslated region (UTR), whole exon regions, whole intron regions and 3’ UTR were obtained from the University of California Santa Cruz genome browser [45] (http://genome.ucsc.edu/). The melanoma enhancer region was obtained from the EnhancerAtlas [46] (http://enhanceratlas.org/index.php). The promoter region was defined as the 3,000 bp around the transcription start site, with 1,500 bp upstream and 1,500 bp downstream. A probe was considered to be located in a given genomic region if its location overlapped with the corresponding region.

For differentially expressed genes, we extracted the probes that were annotated to each gene by the Infinium HumanMethylation 450 BeadChip array from TCGA methylation profiles. Probes were categorized based on their locality on the promoter, 5’ UTR, 3’ UTR, exon or intron region. Then, the mean beta values of the probes in each category were calculated for each patient group. A two-sided Student’s *t* test was used to compare the mean methylation level between each pair of subtypes. A *P* value less than 0.001 was considered significant.

We extracted all the probes in the Infinium HumanMethylation 450 BeadChip array from TCGA methylation profiles, and calculated the number of probes located in each genomic region. For the differentially methylated probes, we also calculated the number of probes located in each genomic region. Fisher’s exact test was used to test the enrichment. Odds ratios and *P* values were obtained, and *P* values were adjusted by the method of Benjamini and Hochberg.

### Gene mutation analysis

We obtained nonsynonymous mutations from the exome-sequencing data of the same cutaneous melanoma patients from the GDC. A two-sided Student’s *t* test was used to evaluate the difference in the number of nonsynonymous mutations between groups. For the analysis of the gene mutation burden, the number of patients harboring the mutated gene in each group was calculated, and the differences between groups were assessed by Fisher’s exact test. A *P* value less than 0.01 was considered significant.

The mutational spectra of all three groups were analyzed with deconstructSigs [47], and the signatures were extracted based on the Wellcome Trust Sanger Institute Mutational Signature Framework [48]. The deconstructSigs package identifies a linear combination of pre-defined signatures that most accurately reconstructs the mutational profile of a tumor sample. Each mutational signature is assigned a calculated weight representing its contribution to the tumor samples, where a higher weight value indicates a greater relative contribution of the signature.

### Survival analysis

For each gene, the top quartile of patients in terms of gene expression was selected as the high expression group, and the bottom quartile of patients was selected as the low expression group. For the three cohorts of melanoma patients from GEO, the top and bottom tertiles of patients in terms of gene expression were chosen as the high and low expression groups, respectively, due to the small sample size. Survival curves were generated by the Kaplan-Meier method, and differences were evaluated by the log-rank (Mantel-Cox) test. OS was calculated from the time of initial diagnosis to death, or was censored to the time when the patient was last known to be alive. All tests were two-sided, and all calculations were performed with R Version 3.3.1 statistical software (R Core Team, Vienna, Austria). The codes are provided in the Supplementary File.

## Supplementary Material

Supplementary Figures

Supplementary Tables

Supplementary Table 1

Supplementary Table 4

## References

[1] Wortsman X. Sonography of the primary cutaneous melanoma: a review. Radiol Res Pract. 2012; 2012:814396. 10.1155/2012/814396 22550586 PMC3328161

[2] Andor N, Graham TA, Jansen M, Xia LC, Aktipis CA, Petritsch C, Ji HP, Maley CC. Pan-cancer analysis of the extent and consequences of intratumor heterogeneity. Nat Med. 2016; 22:105–13. 10.1038/nm.3984 26618723 PMC4830693

[3] Siegel RL, Miller KD, Jemal A. Cancer statistics, 2016. CA Cancer J Clin. 2016; 66:7–30. 10.3322/caac.21332 26742998

[4] Tripp MK, Watson M, Balk SJ, Swetter SM, Gershenwald JE. State of the science on prevention and screening to reduce melanoma incidence and mortality: the time is now. CA Cancer J Clin. 2016; 66:460–80. 10.3322/caac.21352 27232110 PMC5124531

[5] Coupland SE, Lake SL, Zeschnigk M, Damato BE. Molecular pathology of uveal melanoma. Eye (Lond). 2013; 27:230–42. 10.1038/eye.2012.255 23222563 PMC3574255

[6] Rajkumar S, Watson IR. Molecular characterisation of cutaneous melanoma: creating a framework for targeted and immune therapies. Br J Cancer. 2016; 115:145–55. 10.1038/bjc.2016.195 27336610 PMC4947706

[7] Ahmed F, Haass NK. Microenvironment-Driven Dynamic Heterogeneity and Phenotypic Plasticity as a Mechanism of Melanoma Therapy Resistance. Front Oncol. 2018; 8:173. 10.3389/fonc.2018.00173 29881716 PMC5976798

[8] Lawrence MS, Stojanov P, Polak P, Kryukov GV, Cibulskis K, Sivachenko A, Carter SL, Stewart C, Mermel CH, Roberts SA, Kiezun A, Hammerman PS, McKenna A, et al. Mutational heterogeneity in cancer and the search for new cancer-associated genes. Nature. 2013; 499:214–18. 10.1038/nature12213 23770567 PMC3919509

[9] Cancer Genome Atlas Network. Genomic Classification of Cutaneous Melanoma. Cell. 2015; 161:1681–96. 10.1016/j.cell.2015.05.044 26091043 PMC4580370

[10] Metri R, Mohan A, Nsengimana J, Pozniak J, Molina-Paris C, Newton-Bishop J, Bishop D, Chandra N. Identification of a gene signature for discriminating metastatic from primary melanoma using a molecular interaction network approach. Sci Rep. 2017; 7:17314. 10.1038/s41598-017-17330-0 29229936 PMC5725601

[11] Jönsson G, Busch C, Knappskog S, Geisler J, Miletic H, Ringnér M, Lillehaug JR, Borg A, Lønning PE. Gene expression profiling-based identification of molecular subtypes in stage IV melanomas with different clinical outcome. Clin Cancer Res. 2010; 16:3356–67. 10.1158/1078-0432.CCR-09-2509 20460471

[12] Harbst K, Lauss M, Cirenajwis H, Isaksson K, Rosengren F, Törngren T, Kvist A, Johansson MC, Vallon-Christersson J, Baldetorp B, Borg Å, Olsson H, Ingvar C, et al. Multiregion Whole-Exome Sequencing Uncovers the Genetic Evolution and Mutational Heterogeneity of Early-Stage Metastatic Melanoma. Cancer Res. 2016; 76:4765–74. 10.1158/0008-5472.CAN-15-3476 27216186

[13] Rønneberg JA, Fleischer T, Solvang HK, Nordgard SH, Edvardsen H, Potapenko I, Nebdal D, Daviaud C, Gut I, Bukholm I, Naume B, Børresen-Dale AL, Tost J, Kristensen V. Methylation profiling with a panel of cancer related genes: association with estrogen receptor, TP53 mutation status and expression subtypes in sporadic breast cancer. Mol Oncol. 2011; 5:61–76. 10.1016/j.molonc.2010.11.004 21212030 PMC5528272

[14] Chambwe N, Kormaksson M, Geng H, De S, Michor F, Johnson NA, Morin RD, Scott DW, Godley LA, Gascoyne RD, Melnick A, Campagne F, Shaknovich R. Variability in DNA methylation defines novel epigenetic subgroups of DLBCL associated with different clinical outcomes. Blood. 2014; 123:1699–708. 10.1182/blood-2013-07-509885 24385541 PMC3954051

[15] Gevaert O, Tibshirani R, Plevritis SK. Pancancer analysis of DNA methylation-driven genes using MethylMix. Genome Biol. 2015; 16:17. 10.1186/s13059-014-0579-8 25631659 PMC4365533

[16] Netanely D, Avraham A, Ben-Baruch A, Evron E, Shamir R. Expression and methylation patterns partition luminal-A breast tumors into distinct prognostic subgroups. Breast Cancer Res. 2016; 18:74. 10.1186/s13058-016-0724-2 27386846 PMC4936004

[17] Sigalotti L, Covre A, Fratta E, Parisi G, Sonego P, Colizzi F, Coral S, Massarut S, Kirkwood JM, Maio M. Whole genome methylation profiles as independent markers of survival in stage IIIC melanoma patients. J Transl Med. 2012; 10:185. 10.1186/1479-5876-10-185 22950745 PMC3539917

[18] Grzywa TM, Paskal W, Włodarski PK. Intratumor and Intertumor Heterogeneity in Melanoma. Transl Oncol. 2017; 10:956–75. 10.1016/j.tranon.2017.09.007 29078205 PMC5671412

[19] Galon J, Pagès F, Marincola FM, Thurin M, Trinchieri G, Fox BA, Gajewski TF, Ascierto PA. The immune score as a new possible approach for the classification of cancer. J Transl Med. 2012; 10:1. 10.1186/1479-5876-10-1 22214470 PMC3269368

[20] Pagès F, Mlecnik B, Marliot F, Bindea G, Ou FS, Bifulco C, Lugli A, Zlobec I, Rau TT, Berger MD, Nagtegaal ID, Vink-Börger E, Hartmann A, et al. International validation of the consensus Immunoscore for the classification of colon cancer: a prognostic and accuracy study. Lancet. 2018; 391:2128–39. 10.1016/S0140-6736(18)30789-X 29754777

[21] Wu Z, Li X, Qin H, Zhu X, Xu J, Shi W. Ultraviolet B enhances DNA hypomethylation of CD4+ T cells in systemic lupus erythematosus via inhibiting DNMT1 catalytic activity. J Dermatol Sci. 2013; 71:167–73. 10.1016/j.jdermsci.2013.04.022 23706494

[22] Zhu X, Li F, Yang B, Liang J, Qin H, Xu J. Effects of ultraviolet B exposure on DNA methylation in patients with systemic lupus erythematosus. Exp Ther Med. 2013; 5:1219–25. 10.3892/etm.2013.960 23596493 PMC3628076

[23] Nandakumar V, Vaid M, Tollefsbol TO, Katiyar SK. Aberrant DNA hypermethylation patterns lead to transcriptional silencing of tumor suppressor genes in UVB-exposed skin and UVB-induced skin tumors of mice. Carcinogenesis. 2011; 32:597–604. 10.1093/carcin/bgq282 21186298 PMC3066413

[24] Mittal A, Piyathilake C, Hara Y, Katiyar SK. Exceptionally high protection of photocarcinogenesis by topical application of (—)-epigallocatechin-3-gallate in hydrophilic cream in SKH-1 hairless mouse model: relationship to inhibition of UVB-induced global DNA hypomethylation. Neoplasia. 2003; 5:555–65. 10.1016/S1476-5586(03)80039-8 14965448 PMC1502572

[25] Katiyar SK, Singh T, Prasad R, Sun Q, Vaid M. Epigenetic alterations in ultraviolet radiation-induced skin carcinogenesis: interaction of bioactive dietary components on epigenetic targets. Photochem Photobiol. 2012; 88:1066–74. 10.1111/j.1751-1097.2011.01020.x 22017262 PMC3288155

[26] Berwick M, Armstrong BK, Ben-Porat L, Fine J, Kricker A, Eberle C, Barnhill R. Sun exposure and mortality from melanoma. J Natl Cancer Inst. 2005; 97:195–99. 10.1093/jnci/dji019 15687362

[27] Heenan PJ, English DR, Holman CD, Armstrong BK. Survival among patients with clinical stage I cutaneous malignant melanoma diagnosed in Western Australia in 1975/1976 and 1980/1981. Cancer. 1991; 68:2079–87. 10.1002/1097-0142(19911101)68:9<2079::AID-CNCR2820680940>3.0.CO;2-7 1913557

[28] Berwick M, Reiner AS, Paine S, Armstrong BK, Kricker A, Goumas C, Cust AE, Thomas NE, Groben PA, From L, Busam K, Orlow I, Marrett LD, et al, and GEM Study Group. Sun exposure and melanoma survival: a GEM study. Cancer Epidemiol Biomarkers Prev. 2014; 23:2145–52. 10.1158/1055-9965.EPI-14-0431 25069694 PMC4184941

[29] Lindqvist PG, Epstein E, Nielsen K, Landin-Olsson M, Ingvar C, Olsson H. Avoidance of sun exposure as a risk factor for major causes of death: a competing risk analysis of the Melanoma in Southern Sweden cohort. J Intern Med. 2016; 280:375–87. 10.1111/joim.12496 26992108

[30] Gilchrest BA, Eller MS, Geller AC, Yaar M. The pathogenesis of melanoma induced by ultraviolet radiation. N Engl J Med. 1999; 340:1341–48. 10.1056/NEJM199904293401707 10219070

[31] Curtin JA, Fridlyand J, Kageshita T, Patel HN, Busam KJ, Kutzner H, Cho KH, Aiba S, Bröcker EB, LeBoit PE, Pinkel D, Bastian BC. Distinct sets of genetic alterations in melanoma. N Engl J Med. 2005; 353:2135–47. 10.1056/NEJMoa050092 16291983

[32] Zittermann A, Iodice S, Pilz S, Grant WB, Bagnardi V, Gandini S. Vitamin D deficiency and mortality risk in the general population: a meta-analysis of prospective cohort studies. Am J Clin Nutr. 2012; 95:91–100. 10.3945/ajcn.111.014779 22170374

[33] Gnagnarella P, Pasquali E, Serrano D, Raimondi S, Disalvatore D, Gandini S. Vitamin D receptor polymorphism FokI and cancer risk: a comprehensive meta-analysis. Carcinogenesis. 2014; 35:1913–19. 10.1093/carcin/bgu150 25053622

[34] Rawson JB, Sun Z, Dicks E, Daftary D, Parfrey PS, Green RC, Gallinger S, McLaughlin JR, Wang PP, Knight JA, Bapat B. Vitamin D intake is negatively associated with promoter methylation of the Wnt antagonist gene DKK1 in a large group of colorectal cancer patients. Nutr Cancer. 2012; 64:919–28. 10.1080/01635581.2012.711418 22966878 PMC4323165

[35] Goodman AM, Kato S, Bazhenova L, Patel SP, Frampton GM, Miller V, Stephens PJ, Daniels GA, Kurzrock R. Tumor Mutational Burden as an Independent Predictor of Response to Immunotherapy in Diverse Cancers. Mol Cancer Ther. 2017; 16:2598–608. 10.1158/1535-7163.MCT-17-0386 28835386 PMC5670009

[36] Macdonald JB, Dueck AC, Gray RJ, Wasif N, Swanson DL, Sekulic A, Pockaj BA. Malignant melanoma in the elderly: different regional disease and poorer prognosis. J Cancer. 2011; 2:538–43. 10.7150/jca.2.538 22084644 PMC3213678

[37] Tas F, Erturk K. Patient age and cutaneous malignant melanoma: elderly patients are likely to have more aggressive histological features and poorer survival. Mol Clin Oncol. 2017; 7:1083–88. 10.3892/mco.2017.1439 29285379 PMC5740839

[38] Wilkerson MD, Hayes DN. ConsensusClusterPlus: a class discovery tool with confidence assessments and item tracking. Bioinformatics. 2010; 26:1572–73. 10.1093/bioinformatics/btq170 20427518 PMC2881355

[39] Meng C, Helm D, Frejno M, Kuster B. moCluster: Identifying Joint Patterns Across Multiple Omics Data Sets. J Proteome Res. 2016; 15:755–65. 10.1021/acs.jproteome.5b00824 26653205

[40] Anders S, Huber W. Differential expression analysis for sequence count data. Genome Biol. 2010; 11:R106. 10.1186/gb-2010-11-10-r106 20979621 PMC3218662

[41] Wang J, Vasaikar S, Shi Z, Greer M, Zhang B. WebGestalt 2017: a more comprehensive, powerful, flexible and interactive gene set enrichment analysis toolkit. Nucleic Acids Res. 2017; 45:W130–37. 10.1093/nar/gkx356 28472511 PMC5570149

[42] Huang W, Sherman BT, Lempicki RA. Systematic and integrative analysis of large gene lists using DAVID bioinformatics resources. Nat Protoc. 2009; 4:44–57. 10.1038/nprot.2008.211 19131956

[43] Yoshihara K, Shahmoradgoli M, Martínez E, Vegesna R, Kim H, Torres-Garcia W, Treviño V, Shen H, Laird PW, Levine DA, Carter SL, Getz G, Stemke-Hale K, et al. Inferring tumour purity and stromal and immune cell admixture from expression data. Nat Commun. 2013; 4:2612. 10.1038/ncomms3612 24113773 PMC3826632

[44] Tusher VG, Tibshirani R, Chu G. Significance analysis of microarrays applied to the ionizing radiation response. Proc Natl Acad Sci USA. 2001; 98:5116–21. 10.1073/pnas.091062498 11309499 PMC33173

[45] Kent WJ, Sugnet CW, Furey TS, Roskin KM, Pringle TH, Zahler AM, Haussler D. The human genome browser at UCSC. Genome Res. 2002; 12:996–1006. 10.1101/gr.229102 12045153 PMC186604

[46] Gao T, He B, Liu S, Zhu H, Tan K, Qian J. EnhancerAtlas: a resource for enhancer annotation and analysis in 105 human cell/tissue types. Bioinformatics. 2016; 32:3543–51. 10.1093/bioinformatics/btw495 27515742 PMC5181530

[47] Rosenthal R, McGranahan N, Herrero J, Taylor BS, Swanton C. DeconstructSigs: delineating mutational processes in single tumors distinguishes DNA repair deficiencies and patterns of carcinoma evolution. Genome Biol. 2016; 17:31. 10.1186/s13059-016-0893-4 26899170 PMC4762164

[48] Alexandrov LB, Nik-Zainal S, Wedge DC, Aparicio SA, Behjati S, Biankin AV, Bignell GR, Bolli N, Borg A, Børresen-Dale AL, Boyault S, Burkhardt B, Butler AP, et al, and Australian Pancreatic Cancer Genome Initiative; ICGC Breast Cancer Consortium; ICGC MMML-Seq Consortium; ICGC PedBrain. Signatures of mutational processes in human cancer. Nature. 2013; 500:415–21. 10.1038/nature12477 23945592 PMC3776390

