# Supplementary material for: Better prognostic determination and feature characterization of cutaneous melanoma through integrative genomic analysis
Source: Aging (Albany NY). 2019 Jul 18;11(14):5081–107. doi: 10.18632/aging.102099 (PMC6746212; doi:10.18632/aging.102099)
Supplement: Supplementary Figures [file aging-11-102099-s004.pdf]

SUPPLEMENTARY FIGURES

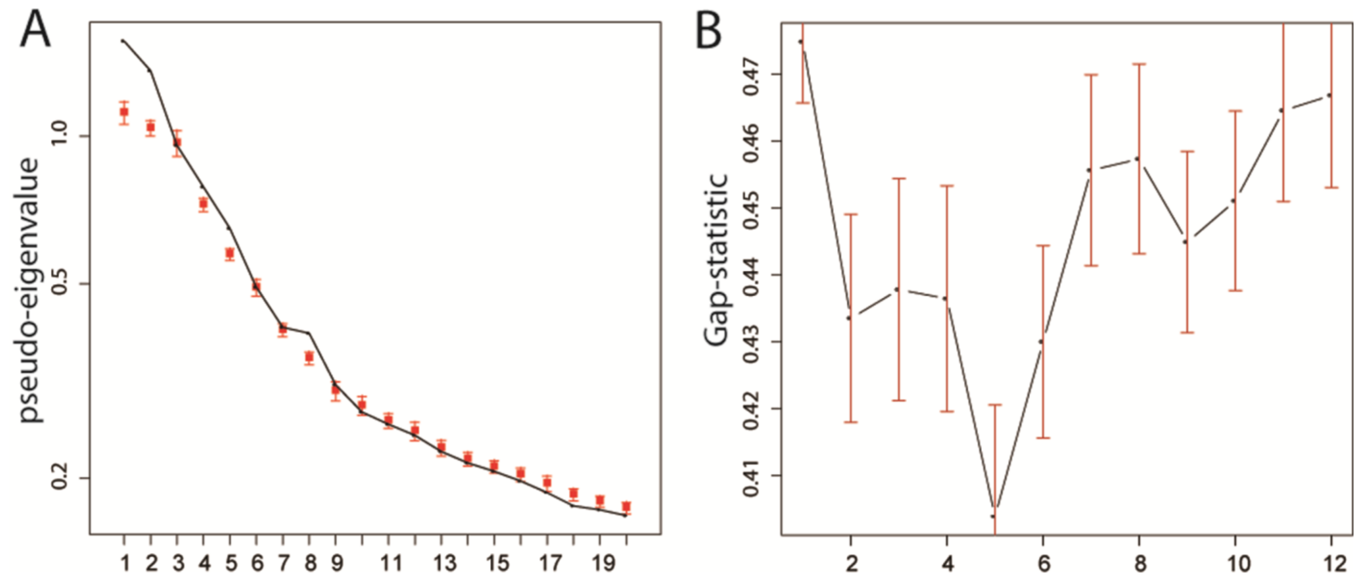

Supplementary Figure 1. Cluster determination in the integrative clustering. (**A**) Permutation test to determine the number of latent variables to include in the clustering. The boxplot displays the eigenvalues for a 100-permutation test; the error bars represent the 95% confidence interval. The results suggested that the top six latent variables accounted for the concordance structures across the datasets. (**B**) Gap statistics with respect to 1 to 12 subclusters indicated that a four-cluster model was the optimal choice.

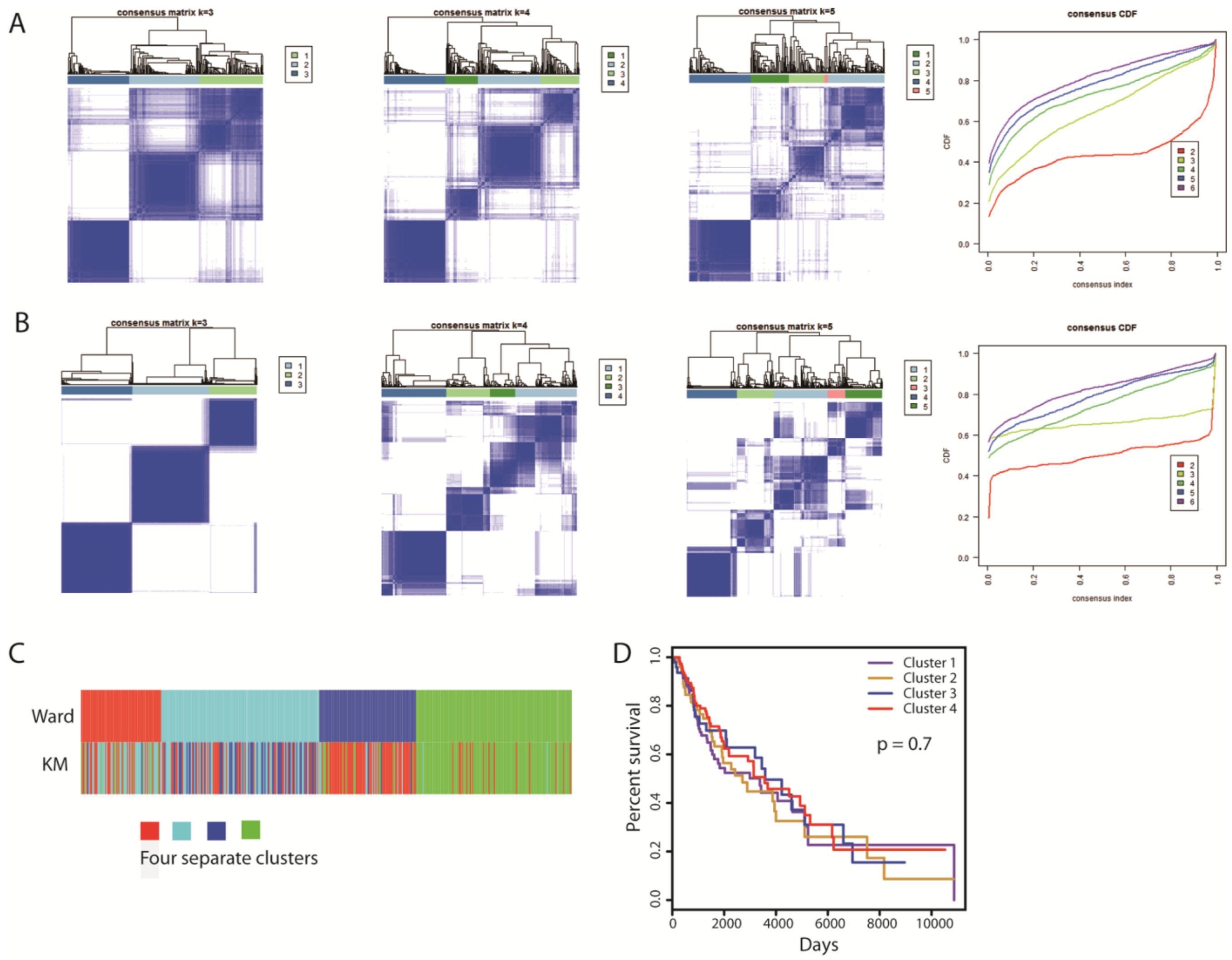

Supplementary Figure 2. Consensus clustering of DNA methylation data. (**A**) Consensus clustering matrices for $k = 3$ to $k = 5$ using the Ward linkage clustering method. The heatmap visually represents the consensus matrix, which is a matrix of sample pairs. Each matrix entry measures the proportion of times that the samples of the pair are clustered together across resampling iterations. The consensus clustering CDF is shown for $k = 2$ to $k = 6$. The optimal number of clusters was determined from the CDF and consensus matrices. (**B**) Consensus clustering was performed by the k-means clustering method. (**C**) The heatmap displays the patient separation in each clustering. Ward: Ward linkage clustering; KM: k-means clustering. (**D**) Kaplan-Meier OS curves are shown for the clusters identified by k-means clustering.

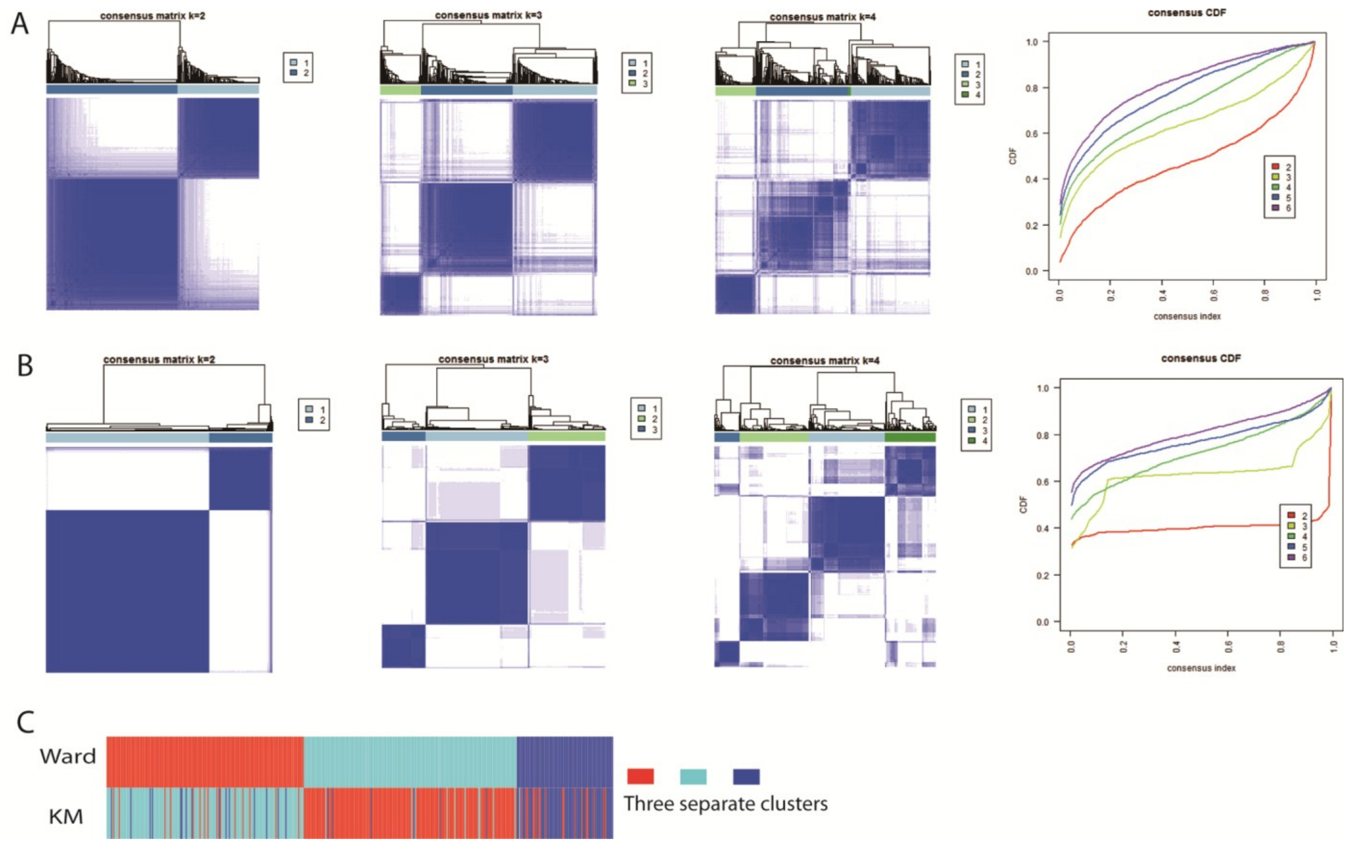

Supplementary Figure 3. Consensus clustering of mRNA-seq data. (**A**) Consensus clustering matrices for k = 2 to k = 4 using the Ward linkage clustering method. The consensus clustering CDF is shown for k = 2 to k = 6. The optimal number of clusters was determined from the CDF and consensus matrices. (**B**) Consensus clustering was performed by the k-means clustering method. (**C**) The heatmap displays the patient separation in each clustering. Ward: Ward linkage clustering; KM: k-means clustering.

	Integrated	Methylation	mRNA	
P1	better	-	-	better
	-	better	-	
	-	-	better	
P2	better	poor	-	neither
	better	-	poor	
	-	better	poor	
P3	better	better	poor	better
	better	poor	better	
	poor	better	better	
P4	better	better	better	→ better

Supplementary Figure 4. Illustration of pooling patients to identify the final prognostic patient grouping. If a patient exhibited better OS in only one clustering, this patient was considered to have a better prognosis, and likewise for patients with poorer OS. When a patient exhibited better OS in one clustering, but poorer OS in other one clustering, the patient was identified as having neither a better nor a poorer prognosis. In other words, a patient was pooled into the better prognostic group only if the patient exhibited significantly better OS in at least two clusterings. A patient exhibiting better OS in all three clusterings was included in the better prognostic group. A similar pooling technique was used for patients with poorer OS.

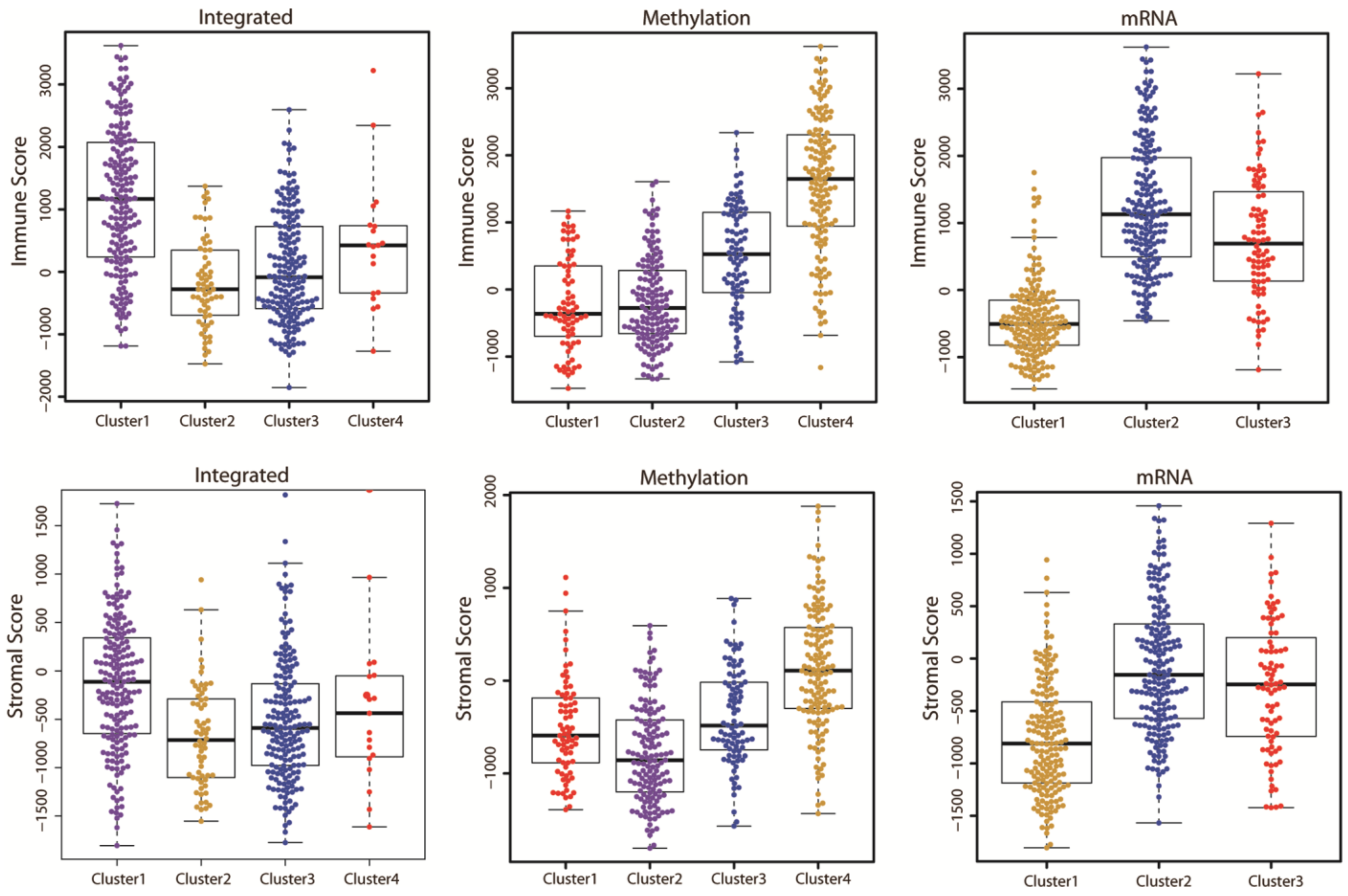

Supplementary Figure 5. Distributions of the immune and stromal scores in the integrated, methylation and mRNA-seq clusterings. Boxplots display the distributions of the immune scores (top panel) and stromal scores (bottom panel) across the three groups in the "Integrated", "Methylation" and "mRNA" clusterings.
