# Supplementary material for: Better prognostic determination and feature characterization of cutaneous melanoma through integrative genomic analysis
Source: Aging (Albany NY). 2019 Jul 18;11(14):5081–107. doi: 10.18632/aging.102099 (PMC6746212; doi:10.18632/aging.102099)
Supplement: Supplementary Tables [file aging-11-102099-s003.pdf]

**Supplementary Table 1. Clinical information on the patients in this study.**

**Supplementary Table 2A. Functions of the genes that were upregulated in the better versus the poorer prognostic group.**

| geneset | description | link | P Value | FDR[1] | overlapGene |
|---|---|---|---|---|---|
| hsa04060 | Cytokine-cytokine receptor interaction - Homo sapiens (human) | http://www.kegg.jp/kegg-bin/show_pathway?hsa04060 | 4.22E-08 | 1.28E-05 | CXCL13;TNFSF13B;CCR8;CSF2RB;IFNG;IL2RA;FASLG;IL7;IL12A;IL15;TNFRSF9;CXCL10;CXCL9;IL21;CXCL11;XCL1;XCL2;CCR2;TNFSF10;IL18RAP;IL18R1 |
| hsa05162 | Measles - Homo sapiens (human) | http://www.kegg.jp/kegg-bin/show_pathway?hsa05162 | 2.43E-06 | 0.000368 | CDK6;DDX58;IFNG;IL2RA;FASLG;IL12A;SH2D1A;PIK3CG;IFIH1;STAT1;TLR2;TNFSF10;CD3G |
| hsa04650 | Natural killer cell mediated cytotoxicity - Homo sapiens (human) | http://www.kegg.jp/kegg-bin/show_pathway?hsa04650 | 1.28E-05 | 0.001295 | FCGR3A;KLRK1;LAT;IFNG;FASLG;KLRC2;KLRC3;KLRD1;LCP2;SH2D1A;PIK3CG;TNFSF10 |
| hsa04062 | Chemokine signaling pathway - Homo sapiens (human) | http://www.kegg.jp/kegg-bin/show_pathway?hsa04062 | 1.73E-05 | 0.001314 | CXCL13;CCR8;DOCK2;PLCB1;GNGT1;CXCL10;ITK;CXCL9;PIK3CG;CXCL11;XCL1;STAT1;XCL2;CCR2 |
| hsa05321 | Inflammatory bowel disease (IBD) - Homo sapiens (human) | http://www.kegg.jp/kegg-bin/show_pathway?hsa05321 | 3.72E-05 | 0.002108 | IFNG;IL12A;IL21;STAT1;STAT4;TLR2;IL18RAP;IL18R1 |
| hsa04620 | Toll-like receptor signaling pathway - Homo sapiens (human) | http://www.kegg.jp/kegg-bin/show_pathway?hsa04620 | 4.17E-05 | 0.002108 | IL12A;CXCL10;CXCL9;TLR8;PIK3CG;CXCL11;STAT1;TLR2;TLR3;CD80 |
| hsa05143 | African trypanosomiasis - Homo sapiens (human) | http://www.kegg.jp/kegg-bin/show_pathway?hsa05143 | 5.43E-05 | 0.002349 | PLCB1;IFNG;FASLG;IL12A;IDO1;VCAM1 |
| hsa04621 | NOD-like receptor signaling pathway - Homo sapiens (human) | http://www.kegg.jp/kegg-bin/show_pathway?hsa04621 | 0.000126 | 0.004759 | TANK;GBP4;GBP5;CYBB;PLCB1;GBP1;GBP2;GBP3;NAIP;STAT1;CASP5;AIM2 |
| hsa05164 | Influenza A - Homo sapiens (human) | http://www.kegg.jp/kegg-bin/show_pathway?hsa05164 | 0.000165 | 0.00557 | DDX58;IFNG;FASLG;IL12A;CXCL10;CIITA;PIK3CG;IFIH1;STAT1;TLR3;TNFSF10;RSAD2 |
| hsa04660 | T cell receptor signaling pathway - Homo sapiens (human) | http://www.kegg.jp/kegg-bin/show_pathway?hsa04660 | 0.000214 | 0.00649 | RASGRP1;LAT;ICOS;IFNG;ITK;LCP2;PIK3CG;PTPRC;CD3G |
| hsa04630 | Jak-STAT signaling pathway - Homo sapiens (human) | http://www.kegg.jp/kegg-bin/show_pathway?hsa04630 | 0.000273 | 0.007531 | CSF2RB;IFNG;IL2RA;IL7;IL12A;IL15;PIK3CG;IL21;STAT1;STAT4;IL27RA |

[1]P value was adjusted using the Benjamini and Hochberg method by WebGestalt.

**Supplementary Table 2B. Functions of the genes that were upregulated in the better versus the poorer prognostic group.**

| Category | Term | P Value | Benjamini[1] | Genes |
|---|---|---|---|---|
| KEGG_PATHWAY | hsa04060:Cytokine-cytokine receptor interaction | 3.26E-08 | 6.14E-06 | IL18R1, IL2RA, IL18RAP, IL7, CXCL9, FASLG, IL15, CXCL11, IL21, CXCL10, CCR8, TNFRSF9, TNFSF10, TNFSF13B, CXCL13, CCR2, IFNG, IL12A, CSF2RB, XCL1, XCL2 |
| KEGG_PATHWAY | hsa05162:Measles | 8.69E-06 | 8.16E-04 | PIK3CG, IFIH1, IL2RA, CD3G, TLR2, FASLG, CDK6, STAT1, DDX58, TNFSF10, SH2D1A, IFNG, IL12A |
| KEGG_PATHWAY | hsa04062:Chemokine signaling pathway | 5.67E-05 | 0.0035478 | PIK3CG, ITK, CXCL9, STAT1, CXCL11, CXCL10, CCR8, GNGT1, DOCK2, CXCL13, CCR2, PLCB1, XCL1, XCL2 |
| KEGG_PATHWAY | hsa04620:Toll-like receptor signaling pathway | 1.94E-04 | 0.0090657 | PIK3CG, CD80, IL12A, TLR2, CXCL9, TLR3, CXCL11, STAT1, TLR8, CXCL10 |
| KEGG_PATHWAY | hsa05321:Inflammatory bowel disease (IBD) | 2.14E-04 | 0.0080151 | IL18R1, STAT4, IL18RAP, IFNG, IL12A, TLR2, STAT1, IL21 |

[1]P value was adjusted using the Benjamini and Hochberg method by DAVID.

**Supplementary Table 3A. Functions of the genes that were downregulated in the better versus the poorer prognostic group.**

| geneset | description | link | P Value | FDR[1] | overlapGene |
|---|---|---|---|---|---|
| hsa00190 | Oxidative phosphorylation - Homo sapiens (human) | http://www.kegg.jp/kegg-bin/show_pathway?hsa00190 | 6.26E-11 | 1.90E-08 | NDUFA11;COX6B1;CYC1;NDUFS7;ATP6;COX3;CYTB;ND1;ND3;NDUFA3;NDUFB7;NDUFC2;NDUFV1;NDUFS8;NDUFA13;ATP5D;ATP5G2;ATP6V0C;ATP6V0A1;ATP6AP1;NDUFB11;COX4I2;ATP6V0D1 |
| hsa01100 | Metabolic pathways - Homo sapiens (human) | http://www.kegg.jp/kegg-bin/show_pathway?hsa01100 | 3.49E-10 | 5.29E-08 | ALG3;PEMT;AGPAT2;CERS1;ADSSL1;NDUFA11;COMT;COX6B1;CYC1;CYP27A1;POLR3H;AHCY;AK1;FAH;ALDH3B1;ALDOA;DOLK;PGLS;GALK1;GAMT;GAPDH;GBA;GPI;GSTZ1;GYS1;IDH3G;ACADS;APRT;ITPKB;NDUFS7;MPST;ATP6;COX3;CYTB;ND1;ND3;NAGLU;NDUFA3;NDUFB7;NDUFC2;NDUFV1;NDUFS8;NME4;NOS3;ACO2;PAFAH1B3;NDUFA13;DCXR;ATP5D;ISYNA1;ATP5G2;PFKL;ATP6V0C;ATP6V0A1;ATP6AP1;DPM3;POLR2I;NDUFB11;NT5M;PYCR1;PCYT2;PYCRL;SMPD2;TKT;TST;TSTA3;TYR;TYRP1;ALG8;HSD17B8;CERS4;PTGES2;FLAD1;PTDSS2;COX4I2;DGAT1;GPAA1;ATP6V0D1;UAP1L1;G6PC3;NAPRT;PPT2 |
| hsa05012 | Parkinson's disease - Homo sapiens (human) | http://www.kegg.jp/kegg-bin/show_pathway?hsa05012 | 4.55E-08 | 4.59E-06 | NDUFA11;COX6B1;CYC1;NDUFS7;ATP6;COX3;CYTB;ND1;ND3;NDUFA3;NDUFB7;NDUFC2;NDUFV1;NDUFS8;NDUFA13;ATP5D;ATP5G2;SEPT5;NDUFB11;COX4I2 |
| hsa05010 | Alzheimer's | http://www.keg | 4.10E-06 | 0.00031 | NDUFA11;COX6B1;CYC1;GAPDH;NDU |

| | | | | | |
|---|---|---|---|---|---|
| | disease - Homo sapiens (human) | g.jp/kegg-bin/show_pathway?hsa05010 | | | FS7;ATP6;COX3;CYTB;NDUFA3;NDUFB7;NDUFC2;NDUFV1;NDUFS8;NDUFA13;ATP5D;ATP5G2;NDUFB11;PSENEN;COX4I2 |
| hsa05016 | Huntington's disease - Homo sapiens (human) | http://www.kegg.jp/kegg-bin/show_pathway?hsa05016 | 6.66E-06 | 0.000404 | CLTB;NDUFA11;COX6B1;CYC1;BBC3;NDUFS7;ATP6;COX3;CYTB;NDUFA3;NDUFB7;NDUFC2;NDUFV1;NDUFS8;NDUFA13;ATP5D;ATP5G2;POLR2I;NDUFB11;COX4I2 |
| hsa03010 | Ribosome - Homo sapiens (human) | http://www.kegg.jp/kegg-bin/show_pathway?hsa03010 | 1.06E-05 | 0.000536 | MRPL21;MRPL2;MRPS2;RPL8;RPL18;RPL18A;MRPL23;RPL28;MRPS12;RPS2;RPS3;RPS10;RPS15;RPS19;RPS28;UBA52 |
| hsa04932 | Non-alcoholic fatty liver disease (NAFLD) - Homo sapiens (human) | http://www.kegg.jp/kegg-bin/show_pathway?hsa04932 | 0.000158 | 0.006841 | NDUFA11;COX6B1;CYC1;NDUFS7;COX3;CYTB;NDUFA3;NDUFB7;NDUFC2;NDUFV1;NDUFS8;NDUFA13;PIK3R2;NDUFB11;COX4I2 |

[1]P value was adjusted using the Benjamini and Hochberg method by WebGestalt.

**Supplementary Table 3B. Functions of the genes that were downregulated in the better versus the poorer prognostic group.**

| Category | Term | P Value | Benjamini[1] | Genes |
|---|---|---|---|---|
| KEGG_PATHWAY | hsa01100:Metabolic pathways | 1.39E-09 | 2.96E-07 | ATP5D, PTGES2, ATP6AP1, CYC1, ALG3, ITPKB, ALG8, FAH, NDUFS7, TYR, IDH3G, NT5M, NDUFS8, GSTZ1, NOS3, ATP6V0D1, PCYT2, AGPAT2, PTDSS2, NDUFB11, ACO2, PFKL, ACADS, COX4I2, NDUFC2, NDUFA13, ALDH3B1, NDUFA11, NAPRT, NME4, PYCR1, PGLS, DGAT1, CYP27A1, FLAD1, MPST, ALDOA, PYCRL, TYRP1, NAGLU, AHCY, NDUFB7, POLR2I, GPAA1, PPT2, ATP5G2, CERS4, COMT, G6PC3, ATP6V0C, GALK1, ISYNA1, CERS1, PEMT, COX6B1, PAFAH1B3, TSTA3, GAPDH, GBA, HSD17B8, ADSSL1, POLR3H, NDUFA3, AK1, TKT, UAP1L1, APRT, DOLK, TST, GPI, NDUFV1, ATP6V0A1, DPM3, GAMT, QPRT, DCXR, SMPD2 |
| KEGG_PATHWAY | hsa00190:Oxidative phosphorylation | 1.08E-06 | 1.15E-04 | ATP5D, NDUFB11, NDUFA3, NDUFB7, ATP6AP1, CYC1, NDUFC2, COX4I2, NDUFA13, ATP5G2, NDUFA11, NDUFS7, ATP6V0C, NDUFV1, NDUFS8, COX6B1, ATP6V0A1, ATP6V0D1 |
| KEGG_PATHWAY | hsa03010:Ribosome | 2.89E-05 | 0.002046409 | MRPL2, RPL18, MRPS12, RPS2, |

| | | | | |
|---|---|---|---|---|
| | | | | RPL28, MRPS2, RPS3, MRPL23, RPS19, MRPL21, RPS28, RPL18A, RPL8, RPS15, RPS10, UBA52 |
| KEGG_PATHWAY | hsa05012:Parkinson's disease | 1.81E-04 | 0.009616315 | SEPT5, ATP5D, NDUFB11, NDUFA3, NDUFB7, CYC1, NDUFC2, COX4I2, NDUFA13, ATP5G2, NDUFA11, NDUFS7, NDUFV1, NDUFS8, COX6B1 |

[1]P value was adjusted using the Benjamini and Hochberg method by DAVID.

**Supplementary Table 4. Association of upregulated gene expression with OS, and validation in three GEO cohorts.**

**Supplementary Table 5A. Functions of the validated prognostic genes.**

| geneset | description | link | P Value | FDR[1] | overlapGene |
|---|---|---|---|---|---|
| hsa04060 | Cytokine-cytokine receptor interaction - Homo sapiens (human) | http://www.kegg.jp/kegg-bin/show_pathway?hsa04060 | 7.15E-07 | 0.000217 | CXCL13;TNFSF13B;CCR8;CSF2RB;IFNG;IL2RA;FASLG;IL7;TNFRSF9;CXCL10;CXCL9;IL18R1 |
| hsa04620 | Toll-like receptor signaling pathway - Homo sapiens (human) | http://www.kegg.jp/kegg-bin/show_pathway?hsa04620 | 1.46E-06 | 0.000222 | CXCL10;CXCL9;TLR8;PIK3CG;STAT1;TLR2;TLR3;CD80 |
| hsa05162 | Measles - Homo sapiens (human) | http://www.kegg.jp/kegg-bin/show_pathway?hsa05162 | 9.53E-06 | 0.000962 | IFNG;IL2RA;FASLG;SH2D1A;PIK3CG;STAT1;TLR2;CD3G |
| hsa04630 | Jak-STAT signaling pathway - Homo sapiens (human) | http://www.kegg.jp/kegg-bin/show_pathway?hsa04630 | 2.85E-05 | 0.002159 | CSF2RB;IFNG;IL2RA;IL7;PIK3CG;STAT1;STAT4;IL27RA |
| hsa04621 | NOD-like receptor signaling pathway - Homo sapiens (human) | http://www.kegg.jp/kegg-bin/show_pathway?hsa04621 | 4.82E-05 | 0.002923 | GBP4;CYBB;GBP1;GBP2;GBP3;NAIP;STAT1;CASP5 |
| hsa04658 | Th1 and Th2 cell differentiation - Homo sapiens (human) | http://www.kegg.jp/kegg-bin/show_pathway?hsa04658 | 7.68E-05 | 0.003878 | LAT;IFNG;IL2RA;STAT1;STAT4;CD3G |
| hsa04640 | Hematopoietic cell lineage - Homo sapiens (human) | http://www.kegg.jp/kegg-bin/show_pathway?hsa04640 | 0.000103 | 0.004469 | IL2RA;IL7;CD1D;CD2;CD3G;CD38 |
| hsa05321 | Inflammatory bowel disease (IBD) - Homo sapiens (human) | http://www.kegg.jp/kegg-bin/show_pathway?hsa05321 | 0.000146 | 0.005387 | IFNG;STAT1;STAT4;TLR2;IL18R1 |
| hsa04660 | T cell receptor signaling pathway - Homo sapiens (human) | http://www.kegg.jp/kegg-bin/show_pathway?hsa04660 | 0.00016 | 0.005387 | RASGRP1;LAT;IFNG;PIK3CG;PTPRC;CD3G |
| hsa04659 | Th17 cell differentiation - Homo sapiens (human) | http://www.kegg.jp/kegg-bin/show_pathway?hsa04659 | 0.000178 | 0.005387 | LAT;IFNG;IL2RA;STAT1;CD3G;IL27RA |

[1]P value was adjusted using the Benjamini and Hochberg method by WebGestalt.

**Supplementary Table 5B. Functions of the validated prognostic genes.**

| Category | Term | P Value | Benjamini[1] | Genes |
|---|---|---|---|---|
| KEGG_PATHWAY | hsa04060:Cytokine-cytokine receptor interaction | 6.06E-07 | 6.96E-05 | TNFRSF9, IL18R1, CCR8, IL2RA, TNFSF13B, CXCL13, IL7, IFNG, CXCL9, CSF2RB, FASLG, CXCL10 |
| KEGG_PATHWAY | hsa04620:Toll-like receptor signaling pathway | 7.40E-06 | 4.26E-04 | PIK3CG, CD80, TLR2, CXCL9, TLR3, STAT1, TLR8, CXCL10 |
| KEGG_PATHWAY | hsa05162:Measles | 3.28E-05 | 0.0012563 | PIK3CG, SH2D1A, CD3G, IL2RA, IFNG, TLR2, FASLG, STAT1 |
| KEGG_PATHWAY | hsa04640:Hematopoietic cell lineage | 3.22E-04 | 0.0092085 | CD38, CD3G, IL2RA, IL7, CD2, CD1D |

[1]P value was adjusted using the Benjamini and Hochberg method by DAVID.

**Supplementary Table 6A. Functions of the genes that were hypomethylated in the better versus the poorer prognostic group.**

| geneset | description | link | P Value | FDR[1] | OverlapGene |
|---|---|---|---|---|---|
| hsa04060 | Cytokine-cytokine receptor interaction - Homo sapiens (human) | http://www.kegg.jp/kegg-bin/show_pathway?hsa04060 | 1.41E-06 | 0.000426 | EDAR;CCR1;CCR4;CCR6;CCR7;CNTFR;CSF2;CSF2RB;CSF3R;IFNLR1;EDA;EGF;EGFR;EPO;FLT3;FLT4;IL17RA;IFNL1;CXCL2;CXCL3;IFNAR2;IFNG;IFNGR2;IL1A;IL2RA;IL2RG;IL4;IL6R;IL7;CXCR1;IL9;CXCR2;IL10RA;IL12RB1;IL13RA1;IL15;IL15RA;IL17A;INHBB;CCL4L1;LIF;LIFR;LTA;LTB;LTBR;OSM;IL21R;IL22;IL23A;PDGFRB;TNFRSF19;PDGFC;ACKR3;CXCL16;IL22RA1;CCL1;CCL3;CCL4;CCL8;CCL11;CCL13;CCL14;CCL15;CCL16;CCL22;CCL23;CXCL5;CXCL12;CXCR5;TGFB2;TNF;TNFRSF1B;TNFSF4;TNFRSF4;IL1R2;TSLP;TNFRSF25;TNFSF14;TNFSF12;TNFSF10;TNFRSF18;TNFRSF10D;OSMR;CD27;TNFRSF8;TNFSF8;CD40 |
| hsa04080 | Neuroactive ligand-receptor interaction - Homo sapiens (human) | http://www.kegg.jp/kegg-bin/show_pathway?hsa04080 | 6.59E-06 | 0.000741 | NPFFR2;GPR83;CHRM3;CHRM4;CHRNA5;CHRNB1;CHRNB2;CHRNB3;CHRNB4;CHRND;CHRNE;CHRNG;ADCYAP1R1;CNR2;ADORA1;CRHR1;CRHR2;ADRA1A;CTSG;ADRA2C;ADRB3;DRD1;APLNR;S1PR1;LPAR1;GABRR3;F2RL1;F2RL2;GABBR1;GABRA1;GABRA2;GABRA4;GABRA6;GABRG3;GALR1;GHSR;GIPR;P2RY10;GLP1R;GLRB;NPBWR1;NPBWR2;MCHR1;GRIA1;GRIA3;GRIA4;GRID1;GRIK2;GRIK3;GRIK4;GRIN2A;GRM6;GRM8;GZMA;HRH2;HTR1B;HTR1E;HTR2A;HTR2C;HTR5A;HTR7;LHCGR;MC3R;MTNR1A;MTNR1B;NMBR;NPY1R;OPRL1;P2RY6;P2RY11;AVPR1B;AVPR2;PTGDR;PTGER2;PTGER3;PTGER4;BDKRB2;SCTR;SSTR1;TACR3;THRB;C3AR1;TSHB;VIPR2;MCHR2;F2RL3;TAAR5;GPR50 |

| | | | | | |
|---|---|---|---|---|---|
| hsa04640 | Hematopoietic cell lineage - Homo sapiens (human) | http://www.kegg.jp/kegg-bin/show_pathway?hsa04640 | 7.33E-06 | 0.000741 | CR1;CR2;CSF2;CSF3R;DNTT;EPO;FLT3;ANPEP;HLA-DMA;HLA-DMB;HLA-DOA;HLA-DPA1;HLA-DRA;IL1A;IL2RA;IL4;IL6R;IL7;ITGA3;ITGA4;ITGA5;ITGB3;MME;TNF;IL1R2;CD1A;CD1B;CD1C;CD1D;CD1E;CD3E;CD5;CD7;CD8A;MS4A1;CD22;CD33;CD34;CD38 |
| hsa04950 | Maturity onset diabetes of the young - Homo sapiens (human) | http://www.kegg.jp/kegg-bin/show_pathway?hsa04950 | 3.64E-05 | 0.002758 | RFX6;NR5A2;HHEX;MNX1;FOXA2;HNF4G;ONECUT1;INS;PDX1;NKX2-2;NKX6-1;NEUROG3;PAX4;PAX6;HNF1B |

[1]P value was adjusted using the Benjamini and Hochberg method by WebGestalt.

**Supplementary Table 6B. Functions of the genes that were hypomethylated in the better versus the poorer prognostic group.**

| Category | Term | P Value | Benjamini[1] | Genes |
|---|---|---|---|---|
| KEGG_PATHWAY | hsa04060:Cytokine-cytokine receptor interaction | 2.11E-06 | 5.98E-04 | OSMR, IL21R, CXCR1, TNFSF14, CXCR2, IL15, TNFSF12, CXCL12, IL17RA, TGFB2, IFNL1, CXCR5, IFNG, CSF3R, IL15RA, CSF2RB, IL13RA1, LTB, IFNGR2, IL1A, LTA, IFNLR1, LTBR, LIFR, CCL4L1, ACKR3, EDAR, CD40, IL6R, IL22, INHBB, OSM, IFNAR2, CCR7, CCR6, CCR4, TNFRSF10D, EDA, CCL1, IL1R2, CSF2, CCL3, TNF, IL22RA1, CXCL5, TNFRSF25, CCR1, CXCL3, CXCL2, CCL8, TNFRSF8, CNTFR, CCL4, TNFRSF4, LIF, IL17A, CCL22, TNFRSF1B, IL12RB1, IL23A, CCL23, IL10RA, TNFRSF18, TNFRSF19, IL2RG, CD27, EPO, IL4, IL2RA, TNFSF4, IL7, IL9, CCL16, CCL15, TNFSF8, CCL11, TSLP, CCL13, TNFSF10, CCL14, CXCL16 |
| KEGG_PATHWAY | hsa04080:Neuroactive ligand-receptor interaction | 6.63E-06 | 9.38E-04 | GPR83, F2RL2, F2RL3, MCHR1, MCHR2, TACR3, THRB, GRIK2, GRIK3, GRIK4, F2RL1, LHCGR, GABBR1, LPAR1, VIPR2, ADORA1, SCTR, HTR1B, S1PR1, GALR1, CHRNA5, TAAR5, HTR5A, GRID1, HTR1E, PTGER2, GABRG3, PTGER3, PTGER4, GZMA, NPBWR1, NPBWR2, GRIN2A, CRHR1, GABRR3, CRHR2, CHRM4, CHRM3, SSTR1, GRM8, HTR7, GRM6, PTGDR, GIPR, GPR50, GLP1R, CTSG, C3AR1, AVPR2, DRD1, ADCYAP1R1, TSHB, BDKRB2, ADRB3, APLNR, P2RY6, HRH2, CNR2, NPFFR2, ADRA2C, MC3R, GABRA2, GLRB, GABRA1, GABRA4, OPRL1, GABRA6, |

| | | | | |
|---|---|---|---|---|
| | | | | GRIA3, GRIA4, NPY1R, P2RY10, P2RY11, GRIA1, AVPR1B, MTNR1B, CHRNB4, ADRA1A, CHRNB3, CHRND, CHRNB2, CHRNB1, NMBR, GHSR, HTR2C, CHRNE, HTR2A, MTNR1A, CHRNG |
| KEGG_PATHWAY | hsa04640:Hematopoietic cell lineage | 4.41E-05 | 0.004152929 | IL1R2, CSF2, TNF, CD8A, MME, ANPEP, ITGB3, DNTT, MS4A1, CSF3R, CD22, CD5, IL1A, EPO, CD7, IL4, CR1, CR2, IL2RA, IL7, FLT3, CD3E, CD1C, CD1B, CD1A, ITGA3, IL6R, ITGA4, CD1E, CD1D, CD38, CD34, ITGA5, CD33, HLA-DRA |

[1]P value was adjusted using the Benjamini and Hochberg method by DAVID.
